# 542. Evaluation of Remdesivir to the Outcomes of Hospitalized Patients with COVID-19 Infection in a Tertiary Care Hospital in Southern India

**DOI:** 10.1093/ofid/ofad500.611

**Published:** 2023-11-27

**Authors:** Jomel Raju, Maria Tom

**Affiliations:** St. Joseph's College of Pharmacy, Cherthala, Pala, Kerala, India; St. Joseph's College of Pharmacy, Cherthala, Pala, Kerala, India

## Abstract

**Background:**

Remdesivir was the only antiviral used in the treatment of COVID-19 in the first wave of the COVID-19 pandemic, following the adaptive COVID-19 treatment trial-1 interim analysis report. However, its use in moderate to critical hospitalized COVID-19 patients continues to be controversial. Therefore, through this nested case-control study in a cohort of hospitalized COVID-19 patients, we aimed to evaluate the benefit of RDV in the treatment of moderate to severe COVID-19.

**Methods:**

In a cohort of 1,531 moderate to critical COVID-19 patients, we retrospectively performed a nested case-control study where 515 patients on Remdesivir were compared to 411 patients with no Remdesivir. Cases and controls were matched for age, sex, and severity. The primary outcome was in-hospital mortality and secondary outcomes were duration of hospital stay, need for intensive care unit (ICU), progression to oxygen therapy, progression to non-invasive ventilation, progression to mechanical ventilation, and duration of ventilation.

**Results:**

The mean age of the cohort was 57.05 + 13.5 years. 75.92% were males. Overall, in-hospital mortality was 22.46% (n = 208). There was no statistically significant difference in all-cause mortality among cases and controls (20.78% vs. 24.57%, p = 0.17). Progression to noninvasive ventilation was lower in the Remdesivir group (13.6% vs 23.7%, p < 0.001), however, progression to mechanical ventilation was higher in the Remdesivir group (11.3% vs 2.7%, p-value < 0.001*). In a subgroup analysis of critically ill patients, the use of Remdesivir lowered mortality (OR 0.32 95% CI: 0.13 - 0.75).

**Conclusion:**

Remdesivir did not decrease the in-hospital mortality in moderate to severe COVID-19 but decreased progression to non-invasive ventilation. Its mortality benefit in critically ill patients needs further evaluation. Remdesivir may be useful if given early in the treatment of patients with moderate COVID-19.

**Disclosures:**

**All Authors**: No reported disclosures

